# Immediate Increase in the Root Mean Square of Successive Differences after Three Bouts of Remote Ischemic Preconditioning: A Randomized Controlled Trial

**DOI:** 10.3390/jcdd11070193

**Published:** 2024-06-26

**Authors:** Charlotte Schöneburg, Benedicta Seyram Amevor, Theresa Bauer, Ivy Boateng, Bright Nsia-Tawia, Nehir Öztürk, Maria-Alexandra Pop, Jan Müller

**Affiliations:** Institute of Preventive Pediatrics, Technical University Munich, 80992 Munich, Germany

**Keywords:** remote ischemic preconditioning (RIPC), autonomic nervous system, heart rate variability, cardioprotection

## Abstract

(1) Background: Remote ischemic preconditioning (RIPC) is an intervention involving the application of brief episodes of ischemia and reperfusion to distant tissues to activate protective pathways in the heart. There is evidence suggesting the involvement of the autonomic nervous system (ANS) in RIPC-induced cardioprotection. This study aimed to investigate the immediate effects of RIPC on the ANS using a randomized controlled trial. (2) Methods: From March 2018 to November 2018, we conducted a single-blinded randomized controlled study involving 51 healthy volunteers (29 female, 24.9 [23.8, 26.4] years). Participants were placed in a supine position and heart rate variability was measured over 260 consecutive beats before they were randomized into either the intervention or the SHAM group. The intervention group underwent an RIPC protocol (3 cycles of 5 min of 200 mmHg ischemia followed by 5 min reperfusion) at the upper thigh. The SHAM group followed the same protocol but on the right upper arm, with just 40 mmHg of pressure inflation, resulting in no ischemic stimulus. Heart rate variability measures were reassessed afterward. (3) Results: The intervention group showed a significant increase in RMSSD, the possible marker of the parasympathetic nervous system (IG: 14.5 [5.4, 27.5] ms vs. CG: 7.0 [−4.3, 23.1 ms], p = 0.027), as well as a significant improvement in Alpha 1 levels compared to the control group (IG: −0.1 [−0.2, 0.1] vs. CG: 0.0 [−0.1, 0.2], p = 0.001). (4) Conclusions: Our results hint that RIPC increases the RMSSD and Alpha 1 parameters showing possible immediate parasympathetic modulations. RIPC could be favorable in promoting cardioprotective or/and cardiovascular effects by ameliorating ANS modulations.

## 1. Introduction

Remote ischemic preconditioning (RIPC) is a therapeutic strategy that applies short periods of ischemia followed by reperfusion, leading to the restoration of blood flow to distant tissues following a temporary reduction. The objective is to trigger protective pathways in various tissues, vascular beds, or organs such as the heart, enhancing their resilience to ischemic damage [1,2,3].

The first successes of RIPC were achieved in the reduction in myocardial infarction during occlusion of the left anterior descending coronary artery when short cycles of occlusion and reperfusion of the left circumflex artery were performed beforehand [4]. By now, RIPC can be readily conducted in a non-invasive manner in clinical settings by alternately inflating and deflating a blood pressure cuff on either the upper arm or thigh. This procedure induces temporary ischemia followed by reperfusion to induce mechanisms such as cardioprotection, including diminishing or preventing myocardial damage [1,5,6].

Despite lacking clarity, it is hypothesized that the signal from a distant tissue such as the heart is transferred through neural, humoral, or systemic signaling channels [1]. The neural hypothesis suggests that molecules produced locally in the ischemic area stimulate an afferent neural pathway, which in turn activates various efferent cardiac pathways that lead to protection of the heart tissue from future (preconditioning) or past (postconditioning) ischemic stimuli [1,2,3,5].

Hence, RIPC might be associated with vagal and sympathetic modulations, leading to variations in heart rate, blood flow, and heart rate variability (HRV), and inducing mild myocardial ischemic stress, all of which contribute to its overall cardioprotective effects [7]. The link between HRV and autonomic control as well as the dominance of vagal over sympathetic regulation of the heart in resting humans was demonstrated by the decrease in RR variability after vagal blockade with high-dose atropine [8,9]. Furthermore, the RR mean interval offers insights into a balance between sympathetic and vagal neural activities. Additionally, the degree of RR interval variability around its mean may reflect the equilibrium of spontaneous fluctuations in vagal and sympathetic neural activities, which can be referred to as vagal and sympathetic modulations [10].

The possible involvement of the ANS was initially recognized through experiments where pretreatment with the ganglionic blocker hexamethonium hindered the cardioprotective effects induced by RIPC [11,12,13].

In an experimental study with rats, researchers investigated whether increased systemic efferent vagal tone, largely mediated by intrinsic cardiac ganglia, was associated with cardioprotection [14]. Furthermore, a study analyzing the effects of intermittent limb ischemic changes observed alterations in cardiac output and organ blood flow as additional effects of RIPC where the ANS may be involved [15]. A recent pilot study indicated that the RIPC procedure modulated both the parasympathetic and sympathetic branches of the ANS in healthy adults measured with electrocardiography (ECG) [16]. Additionally, another study investigated the effects of RIPC on HRV and stress resistance. The HRV values in healthy young volunteers confirmed possible evidence of the involvement of the ANS in experimental models of RIPC-induced protection [17].

Cardioprotection is triggered by preconditioning in two phases (early and late RIPC). In the early phase of RIPC, also known as the first window of protection, the onset of protective effects occurs promptly following the RIPC stimulus and endures for a brief period, ranging from a few minutes to several hours. In the late or delayed phase of preconditioning (second window), the protective mechanisms are initiated 24 hours after the RIPC stimulus and persist for an extended duration, spanning from 48 to 72 hours [4,18,19,20].

On the one hand, previous studies have shown the early effects of RIPC regarding protection against ischemia-reperfusion injury, endothelial function, or the activation of the neurogenic pathway [19,21,22]. On the other hand, several studies have demonstrated no short-term effects of RIPC on blood pressure, heart rate, and arterial stiffness [6,23]. Thus, the early protective effects of RIPC on the ANS in healthy individuals have not been adequately established. 

Therefore, the objective of this study is to investigate the immediate effects of a three-cycle RIPC intervention on the ANS via HRV measurement in a randomized controlled trial.

## 2. Materials and Methods

### 2.1. Study Subjects

From March 2018 to November 2018, we enrolled a total of 51 healthy volunteers (29 female, with an average age of 24.9 [23.8, 26.4] years) for participation in this single-blinded randomized controlled trial conducted at our institution. Table 1 outlines the study characteristics and baseline measurements of all participants.

To ensure standardization, all measurements were performed between 8:00 and 10:00 a.m. in the morning. The participants met specific inclusion criteria: being free from infections, in a sober state, and abstaining from alcohol and tobacco for the preceding twelve hours.

The study was conducted in accordance with the Declaration of Helsinki (revision 2008) and followed the Good Clinical Practice guidelines. The study protocol received approval from the local ethical board at the Technical University of Munich under project number 209/18S. All participants provided written informed consent, granting permission for the anonymous publication of their data.

### 2.2. Assessment of the Autonomous Nervous System

The function of the autonomic nervous system (ANS) was quantified by a non-invasive heart rate variability (HRV) measurement with participants in a supine position, in a darkened and acclimatized room, using the VNS Analysis Professional Software (Commit GmbH, Liebenburg, Germany). After a 5-min resting phase, 260 adjacent heartbeats were recorded. 

To identify time-based HRV parameters, the time between the successive R waves is calculated by registering the R waves in the QRS complex. RR intervals were recorded with an established high-resolution HRV chest strap system with bipolar chest leads. Patients were instructed to pursue a relaxed state and to breathe normally. In this study, no breathing frequency was specified. In comparison to high-time resolution electrocardiogram (EKG) systems, the Analysis Professional Software used shows very good validity with a measurement accuracy of ±1 ms [24] and is therefore in line with recent HRV measurement guidelines [25]. With a guarantee of quality measurement, the accuracy of the parameters’ computation is adjusted regularly using the scientific reference software Kubios HRV 2.1 [26]. This validation of the software ensures a reliable measurement device for HRV and complies with high scientific standards. Moreover, the VNS software had a built-in arrhythmia detection algorithm, and all 260 beat-to-beat intervals were free from arrhythmia. 

For statistical analysis, the root mean square of successive differences (RMSSD), standard deviation of normal-to-normal beats (SDNN), stress index (SI), Alpha 1, and the heart rate were used.

In detail, RMSSD quantifies the variability in the intervals between successive heartbeats. Specifically, RMSSD reflects the magnitude of change in heart rate from one beat to the next, providing insights into parasympathetic nervous system modulations. Higher RMSSD values typically indicate greater parasympathetic influence and are associated with better cardiovascular health and lower stress levels. SDNN stands for standard deviation of NN intervals. It is another time-domain measure of heart rate variability (HRV) that quantifies the overall variability in the intervals between successive normal-to-normal (NN) heartbeats, regardless of the sequence order. SDNN reflects both sympathetic and parasympathetic influences on heart rate.

The key difference between RMSSD and SDNN lies in their focus and sensitivity to different aspects of HRV. RMSSD specifically captures short-term variability in heart rate, primarily influenced by parasympathetic modulations. It emphasizes beat-to-beat changes and is particularly sensitive to respiratory-induced fluctuations in heart rate. On the other hand, SDNN reflects overall HRV, integrating both short-term and long-term variations, and is influenced by both sympathetic and parasympathetic activities. While RMSSD is more sensitive to parasympathetic modulation, SDNN provides a broader view of autonomic nervous system function.

Baevsky’s stress index (SI) is the geometric HRV measure that reflects cardiovascular stress, with high values indicating significant sympathetic activation and reduced variability [27], while Alpha 1 refers to a parameter derived from detrended fluctuation analysis (DFA). It quantifies the short-term fractal-like correlation properties of heart rate dynamics and determines the quality of regulation and how the individual regulatory systems work together. This can be illustrated in an example of a measurement under synchronized breathing. If a measurement is carried out under a determined breathing rhythm, a respiratory sinus arrhythmia occurs, which can be clearly recognized in the rhythmogram. The signal looks very uniform, which means that there is more stability in the system. The regulatory systems work very closely together, resulting in so-called coherence. The Alpha 1 value therefore inevitably increases during clock breathing. This should therefore not be viewed negatively, but rather shows that the systems can enter into coherence.

### 2.3. Intervention 

After resting for 5 minutes in a supine position, the participants received an HRV measurement, as previously described [6]. Afterward, they were simply randomized into an intervention (25) or SHAM (26) group by the principal investigator. The intervention group received an RIPC protocol of 3 cycles of 5 minutes each, with occlusion to 200 mmHg with a special blood pressure BP cuff on the right thigh, followed by 5 minutes of reperfusion. More details on RIPC and the protocols used can be found in the review from Heusch and colleagues [1].

The SHAM procedure consisted of a pressure cuff inflated on the right upper arm for the same periods as the RIPC intervention, but only to 40 mmHg, as it has been shown to mimic occlusion but not limit blood flow. Study participants were thus led to believe that this study was a comparison between different occlusion techniques on the thigh and the upper arm.

**Figure 1:**
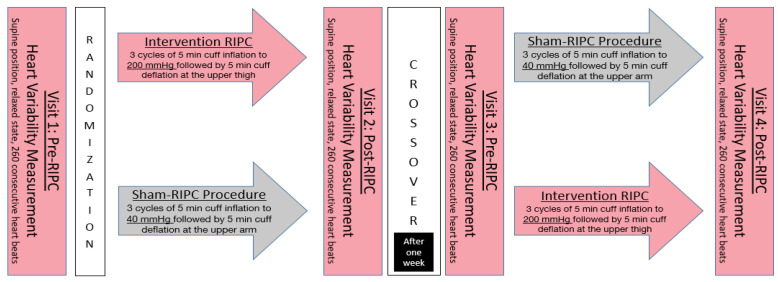
Patient inclusion and study protocol.

Directly after this 30-minute intervention or SHAM procedure, a reassessment of HRV measures was conducted. After a mean of 6.8 ± 1.0 days, a crossover was performed, and participants were assigned to the other group and underwent the procedure again (Figure 1).

### 2.4. Data Analyses

Due to a high skewness and spread of the RMSSD (IG: 14.5 [5.4, 27.5] ms vs. CG: 7.0 [−4.3, 23.1] ms), it was assumed that the data were not normally distributed. 

Thus, a paired Mann–Whitney U test was used as a non-parametric test method to compare median differences pre- and post-test between both intervention and control groups. A difference between control and intervention groups was considered statistically significant at a *p*-value < 0.05 with the respective confidence intervals indicated. For statistical analyses, the software R version 2022.07.1 was used. R-Package “ggstatsplot” was applied to illustrate the results in figures.

## 3. Results

After three bouts of RIPC, there was a significant increase in root mean square of successive differences (RMSSD) in the intervention group from 43.0 ms to 57.5 ms, and an increase in the control group from 41.5 ms to 48.5. However, the increase was significantly higher in the intervention group compared to the control group (IG: 14.5 [5.4, 27.5] ms vs. CG: 7.0 [−4.3, 23.1] ms, p = 0.024). Moreover, in comparison to the control group, Alpha 1 remained stable in the intervention group with a value of 1.0, whereas we noticed a small decline in the CG from 1.0 to 0.9. Group comparisons also showed a significant difference between the groups (IG: 0.0 [−0.1, 0.2] vs. CG: −0.1 [−0.2, 0.1], p = 0.008). 

**Figure 2:**
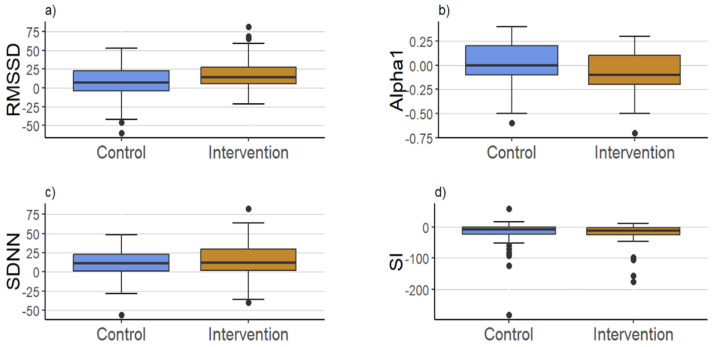
Boxplots of the four heart rate variability parameters (**a**) RMSSD, (**b**) Alpha 1, (**c**) SDNN, (**d**) SI showing pre- and post-test differences.

There were no significant changes in stress index (SI), standard deviation of normal-to-normal beats (SDNN), and heart rate between groups according to the intervention. For an illustration of the results, see Figure 2.

## 4. Discussion

In this randomized controlled trial, the possible role of remote ischemic preconditioning (RIPC) in vagal and sympathetic modulations was assessed by analyzing several heart rate variability (HRV) parameters, such as the root mean square of successive differences (RMSSD), the standard deviation of normal-to-normal beats (SDNN), stress index (SI), and Alpha 1, before and after the procedure. Among the measured and analyzed parameters, only those that were short-term, which indicated parasympathetic modulations of HRV parameters, such as RMSSD and Alpha 1, were significantly affected after three bouts of RIPC in healthy individuals.

HRV is an individualized metric in which a higher variability represents higher adaptability to changing internal and external conditions [28]. Apart from that, physiological changes relevant to the individual’s health may be indicated by a low HRV, which can reflect the insufficient adaptability of the autonomic nervous system. While heart rate variability can exhibit significant variations due to circadian rhythms [29], the RIPC procedure in this study was consistently administered at the same time frame (between 8 and 10 a.m., with a total procedural duration of 30 minutes). Consequently, the daily fluctuations may not be a predominant factor contributing to the observed variations in heart rate parameters. 

This study provides a comprehensive assessment of vagal and sympathetic modulations, indicating the regulation of the autonomic nervous system, by recording several HRV values, such as the RMSSD, SDNN, SI, and Alpha 1. The short-term index Alpha 1 of the trend-adjusted fluctuation analysis is, next to the others, an established parameter that is prognostically relevant for characterizing the regulatory capacity of the ANS [30].

Our findings are in line with other studies providing evidence that ANS is involved in RIPC-induced protection through increased RMSSD levels, not only in healthy individuals, but also in patients with heart disease [16,31,32]. As an example, in one pilot study with healthy adults with non-linear HRV parameters, SD2 was significantly increased by the RIPC procedure. An elevation in SD2 levels implies an activation of both the parasympathetic and sympathetic branches [16]. In addition, RIPC studies in patients with heart failure and stroke showed the improvement of the autonomic function of the ANS and the patients’ quality of life [31,32].

In contrast, in a self-controlled interventional study, RMSSD did not differ significantly between the control and RIPC group in healthy adults [33]. The insignificance of RMSSD in the study may be because RIPC was performed on individuals only once, which could not lead to changes in the ANS modulations. However, conducting multiple cycles of RIPC may result in protective effects, as suggested in previous studies [34]. 

Nonetheless, the efficacy of a single acute session of remote ischemic preconditioning (RIPC) remains contentious. Some studies in humans have demonstrated the potential positive effects of remote ischemic preconditioning by suppressing sympathetic elevation and oxidative stress, as well as enhancing reactive hyperemic response followed by ischemia-reperfusion injury in healthy individuals [19,35]. Among patients with myocardial infarction, a decrease was observed in myocardial tissue damage [36]. However, on the other hand, studies suggest that a single bout of RIPC has a negligible impact on autonomic function in young healthy individuals and does not affect cerebrovascular function in the elderly [37]. Another study using RIPC in healthy individuals showed that a single bout of RIPC was not sufficient to alter HRV indices when tested at multiple time points. However, they realized that repeated bouts of RIPC induced changes in HRV [38]. Their findings were supported by both power spectral density and symbolic dynamic analyses, indicating a shift in the sympatho-vagal balance toward greater parasympathetic activity following two weeks of RIPC. The data derived from their study also showed that no second window effect was recorded on HRV after a single bout of RIPC was performed [38].

Additionally, the effectiveness of RIPC appears to be diminished in certain clinical conditions, such as type 2 diabetes mellitus [39], whereas obesity did not negate the beneficial impacts of RIPC in Asian obese men [40]. These discrepancies, particularly in human studies, might be partially attributed to variations in health profiles or cardiovascular disease risk factors among participants [41].

It is a widely recognized fact that cardiovascular diseases often lead to increased sympathetic modulations and suppressed parasympathetic fluctuations. The Alpha 1 parameter is influenced by cardiovascular diseases, leading to elevated sympathetic modulations and possible decreased parasympathetic activity [42]. Previous research has indicated that physical training could have a significant impact on improving exercise capacity and mitigating autonomic dysfunction in chronic heart failure by augmenting parasympathetic activity [43,44]. Experimental studies have also suggested that increased parasympathetic activity may reduce the risk of mortality [45] and sudden cardiac death, including instances of ventricular fibrillation [46,47]. Consequently, parasympathetic stimulation could be of critical importance in the management of heart disease. This makes it even more important to extend adequate implementation and treatment to different clinical scenarios.

### Limitations

The primary limitations of this study include its small sample size and relatively young participants. Specifically, this study is a randomized controlled trial involving 51 healthy participants aimed at investigating the potential involvement of the autonomic nervous system in RIPC and delving deeper into its underlying mechanisms in protecting distant organs. Future studies need to include a larger number of subjects to provide a more comprehensive assessment of the role of the autonomic nervous system.

Respiratory sinus arrhythmia has a crucial impact on the parameters of HRV. Some studies standardize the breathing frequency for this reason. However, in this case, we have decided not to standardize it because imposing a rigid specification of the breathing frequency can be uncomfortable for some individuals, potentially leading to changes in HRV. Since we have pre- and post-settings, we have chosen to let the individuals breathe arbitrarily, considering that they will do the same during the post-measurement, thus ensuring a more comparable measurement setup.

The VNS software had a built-in arrhythmia detection algorithm; however, we could not completely rule out the possibility that some arrhythmias or ectopic beats may have biased the analysis. Alpha 1 derived from DFA might need longer sequences for its safe computation and interpretation. The results for Alpha 1 should be viewed with caution, as the difference is very small, even if significant. Nevertheless, it cannot be definitively assumed that this results in clinical relevance.

The RR variability markers, as indicators of autonomic modulation using RMSSD, should be interpreted with caution. In particular, if the RR mean varies between groups and experimental conditions and thus the actual role of altered neuronal modulation is not justified, it is recommended to check for potential variations in the LF (low-frequency)/HF (high-frequency) ratio before concluding that RR variability indices in absolute units indicate modifications of autonomic control [48].

Ergoreceptors in muscles detect ischemia and signal this to the central nervous system, which increases sympathetic outflow, raising blood pressure and heart rate. A direct measurement of the baroreflex, including blood pressure and heart rate, would have been useful to assess changes in HRV more precisely and to be able to discuss the pathophysiological effects, especially after reperfusion.

The modulation of the autonomic nervous system induced by RIPC was solely evaluated during the RIPC procedure and in brief intervals before and after it. Consequently, further investigations are warranted for exploring the possibility of a secondary window for autonomic nervous system modulation, particularly 24 h post-RIPC procedure. Additionally, employing more frequent RIPC sessions (such as daily) may potentially enhance the observed effects. 

## 5. Conclusions

Our results suggest that RIPC increases the RMSSD and Alpha 1 parameters, indicating possible immediate parasympathetic modulation changes. These results may point out that ANS involvement could be one of the mechanisms for achieving RIPC therapeutic effectiveness. In conclusion, RIPC may favorably promote cardioprotective effects by ameliorating ANS modulations.

## Figures and Tables

**Table 1:** Study characteristics of the 51 participants.

Variable	Median [Q1, Q3]
Gender (female)	29 (56.9%)
Age (years)	24.9 [23.8, 26.4]
BMI	21.9 [20.3, 23.4]
Heart rate (beats/min)	66.8 [59.6, 72.3]
RMSSD (ms)	50.3 [33.6, 84.5]
SDNN (ms)	60.5 [46.7, 83.5]
SI	28.9 [19.4, 54.6]
Alpha 1	1.00 [0.80, 1.2]

BMI = body mass index; RMSSD = root mean square of successive differences; SDNN = standard deviation of normal-to-normal beats; SI = stress index.

## Data Availability

The dataset can be requested from the corresponding author.
